# Household Responses to School Closure Resulting from Outbreak of Influenza B, North Carolina 

**DOI:** 10.3201/eid1407.080096

**Published:** 2008-07

**Authors:** April J. Johnson, Zack S. Moore, Paul J. Edelson, Lynda Kinnane, Megan Davies, David K. Shay, Amanda Balish, Meg McCarron, Lenee Blanton, Lyn Finelli, Francisco Averhoff, Joseph Bresee, Jeffrey Engel, Anthony Fiore

**Affiliations:** * Centers for Disease Control and Prevention, Atlanta, Georgia, USA; † North Carolina Department of Health and Human Services, Raleigh, North Carolina, USA; ‡ Toe River Health District, Burnsville, North Carolina, USA

**Keywords:** Influenza, human, disease outbreaks, schools, research

## Abstract

School closure is a proposed strategy for reducing influenza transmission during a pandemic. Few studies have assessed how families respond to closures, or whether other interactions during closure could reduce this strategy’s effect. Questionnaires were administered to 220 households (438 adults and 355 children) with school-age children in a North Carolina county during an influenza B virus outbreak that resulted in school closure. Closure was considered appropriate by 201 (91%) households. No adults missed work to solely provide childcare, and only 22 (10%) households required special childcare arrangements; 2 households incurred additional costs. Eighty-nine percent of children visited at least 1 public location during the closure despite county recommendations to avoid large gatherings. Although behavior and attitudes might differ during a pandemic, these results suggest short-term closure did not cause substantial hardship for parents. Pandemic planning guidance should address the potential for transmission in public areas during school closure.

## Abstract 2

Parents accepted school closure during an outbreak, but children’s presence in other public settings has implications for pandemic planning.

Human influenza pandemics have occurred 3 times in the past century and are assumed to be recurring biologic events (*1*). Preparation for the next influenza pandemic has become a major focus of public health activities. Use of vaccines containing antigens matched for a pandemic influenza A strain is the best control measure for reducing illness and death during a pandemic (*1*,*2*). However, specific vaccines will take >4–6 months to be produced once a pandemic strain is identified. Additionally, sufficient doses of antiviral drugs might not be available to supply treatment and chemoprophylaxis needs (*3*). As a result, a variety of nonpharmaceutical interventions (NPIs) have been proposed by US federal agencies (*4*) and the World Health Organization (*5*) to help mitigate the impact of a pandemic while vaccines against the pandemic strain are being produced. NPIs that have been identified as potential mitigation strategies include voluntary isolation of case-patients, voluntary quarantine of contacts of cases, and social distancing of children and adults.

School-age children have the highest attack rates during typical seasonal influenza outbreaks and play a central role in sustaining influenza transmission (*6*). Children are being disproportionately affected by the avian influenza A virus (H5N1) that is currently circulating in many countries (*7*). School attendance during the 1957 epidemic was thought to amplify the transmission of virus in Japan (*8*). Proposed mitigation strategies have thus focused on this age group as a means of reducing transmission. School closure lasting 4–12 weeks has been recommended as an option to distance children and decrease transmission (*4*). Several modeling studies have suggested that school closure might reduce peak attack rates and overall clinical attack rates, especially if combined with other strategies, including voluntary isolation and quarantine of sick persons and their contacts (*9*) or household-based antiviral prophylaxis and quarantine (*10*). However, few data are available to address whether school closure can actually reduce the transmission of influenza viruses among susceptible children or their family contacts (*11*).

Prolonged school closures might have adverse social and economic effects (*12*,*13*). For example, some parents will likely stay home to care for children, resulting in lost family income, as well as adverse effects on businesses. Children from families of lower socioeconomic status may rely on their schools for the National School Lunch Program, a federally assisted meal program that provides meals and snacks to children who qualify (*13*). To date, no study has evaluated parental attitudes or responses to school closures during a seasonal influenza outbreak.

In late October, 2006, a rural county in North Carolina experienced an influenza B virus outbreak that resulted in a sudden increase in student and school staff absenteeism. School officials closed all 9 schools in the county on November 2, and schools remained closed through November 12. These events provided an opportunity to evaluate the response of families with schoolchildren to closing schools and to observe the frequency of children’s excursions to public places during the school closure.

## Methods

### Detection of and Response to the Outbreak

**Figure 1:**
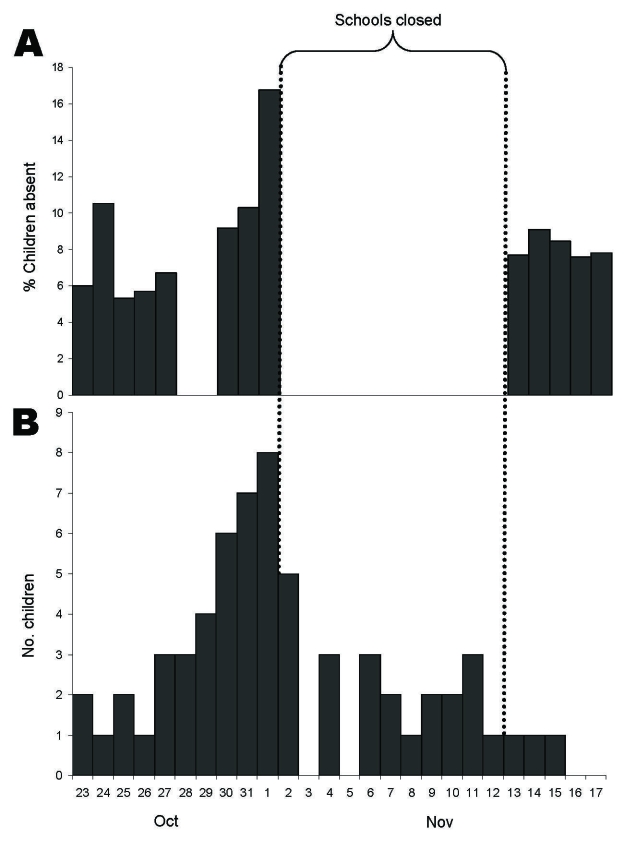
A) Percentage of schoolchildren absent from public schools, by date, and B) total number of children surveyed with influenza-like illness, by date of illness onset, Yancey County, North Carolina, October 23–November 17, 2006.

Yancey County, North Carolina, had an estimated population in 2006 of 18,421, of whom ≈21% were <18 years of age, and an estimated 7,472 households in 2000. This county is located in the western part of the state in the Appalachian Highlands on the Tennessee border. From October 26 through November 1, 2006, school officials observed a marked increase in the number of student and employee absences in the 9-school system. Many absences were attributed to influenza-like illness (ILI) among children and staff. Two elementary schools were particularly affected, with absentee rates among students increasing from 4% and 8.8% on October 26 to 34% and 32%, respectively, on November 1. Using commercial rapid antigen detection techniques, a local clinic identified influenza in 29 patients on October 31. The North Carolina Public Health Laboratory subsequently confirmed the presence of influenza B virus in samples that were submitted for viral culture from 7 of 8 children. On November 1, with 429 children (17% of schoolchildren enrolled in all 9 schools; Figure 1, panel **A**) and 38 (10%) of the staff absent, school officials closed all the county’s public schools because of unmet staffing needs.

In an increased effort to vaccinate residents in response to the outbreak, influenza vaccine clinics were established at the county health department. A reverse 911 call was issued to county residents by the health department and county government on November 1. The reverse 911 system is a notification system that enables town officials to deliver telephone messages during an emergency to specific groups of persons on the basis of location. County residents were given the following message by telephone: “This is a message from Yancey County Health Department and Yancey County Government. Due to increasing cases of influenza, residents of Yancey County are being asked to avoid large gatherings. Also, please wash hands frequently, cover coughs, and avoid contact with sick individuals. For more recorded information call ...”

### Household Survey

A total of 1,750 households had children enrolled in the public school system. To evaluate the response of Yancey County residents with children in the public school system to the influenza outbreak and school closings, a random sample of these households was contacted by telephone on November 16–18. A standard questionnaire was used. A parent or legal guardian from each household was asked to provide information about his or her child’s (or children’s) activities during the school closure (November 2–12, 2006), special arrangements that had to be made for child care, and attitudes toward the closure. This household respondent was also asked to provide demographic information on the household and answer questions about how school closure affected his or her own employment and daily routines, and those of any other adults in the household. Respondents were asked their perceptions of the likelihood that a child might be infected with influenza virus and the likelihood that an infected child would require hospitalization. Parents with children who had been ill at some time since October 15, 2006, were asked about clinical manifestations of each child’s illness. ILI was defined as having a fever (objective or subjective) and either a cough or sore throat in the absence of a known cause other than influenza.

Four hundred telephone numbers were randomly selected from a list of all households with children enrolled in public schools, with the intent of obtaining >200 completed surveys, which would represent >10% of the households and >10% of children enrolled in public schools. Families were called at various times of the day and evening both during the week and on the weekend from November 16–18. Information was collected on each school-age child living in the household. To control for the effect of family, 1 child was randomly selected from each household. Statistical analysis was performed by using SAS version 9.1 (SAS Institute, Cary, NC, USA).

## Results

Interviewers called households over a 3-day period until a minimum of 200 surveys were completed. Two hundred twenty (97%) of those contacted completed the survey; 8 (3%) households refused. The 220 households surveyed included 438 adults (≈3% of the adult population in the county) and 355 school-age children (≈14% of all children enrolled in the public schools, or 9.4% of all children <18 years of age in the county). The percentage of children surveyed from any 1 school ranged from 9% to 21%. The percentage of children in each grade who were surveyed ranged from 8% of third-graders to 20% of ninth-graders. Characteristics of the household and children in the survey are shown in Table 1. Thirty-seven (17%) of the 220 households had only 1 adult (lower than the national rate of single-parent homes, which is 27%). Children from 87 (41%) of 212 responding households were reported as receiving free or reduced-cost lunches through the National School Lunch Program. This finding is slightly lower than the percentage of children reported as enrolled in the county (51%) or the state (48%) in 2005 (*14*), but approximately the same as the national (37.8%) percentage of children eligible for the program (*15*).

**Table 1:** Characteristics of children and households surveyed, Yancey County, North Carolina, 2006*

Characteristic	Value
Households (N = 220)	
Single-adult home	37 (17)
Two-adult home	145 (66)
Three- or four-adult home	38 (17)
Children in home receive free/reduced-cost lunch† (n = 212)	87 (41)
All adults employed outside the home	118 (54)
Children (N = 355)	
Male	177 (50)
White, non-Hispanic	344 (97)
Median age, y (range)	12 (5–19)

*Values are no. (%) unless otherwise indicated. †Free and reduced-cost lunches provided through the National School Lunch Program.

One hundred thirty (37%) of 355 surveyed schoolchildren were ill on >1 days from October 23 through November 15. Among children who attended elementary schools, 50% were ill during this period, compared with 26% and 28% of surveyed middle and high school-age children, respectively (Table 2). Among children who were reported ill, 66 (51%) met the case definition for ILI. The reported dates for ILI symptom onset started October 20 and peaked on November 1, 2006, the day before schools closed (Figure 1, panel **B**). A total of 78 (22%) children reportedly received influenza vaccine for that season as of November 15. Sixty-three (81%) of these children received it after schools closed (November 2 or later).

**Table 2:** Prevalence of child illness reported by parents by school type, Yancey County, North Carolina, 2006

School type	No. (%) surveyed of all public schoolchildren	No. (%) ill of those surveyed
Elementary	136 (12)	68 (50)
Middle	86 (14)	22 (26)
High	128 (16)	36 (28)

Ninety-nine (45%) household respondents thought it was very possible that their child could get influenza from another person. However, only 15 respondents (7%) felt it was very possible that their child would need hospitalization if they became infected with influenza.

**Figure 2:**
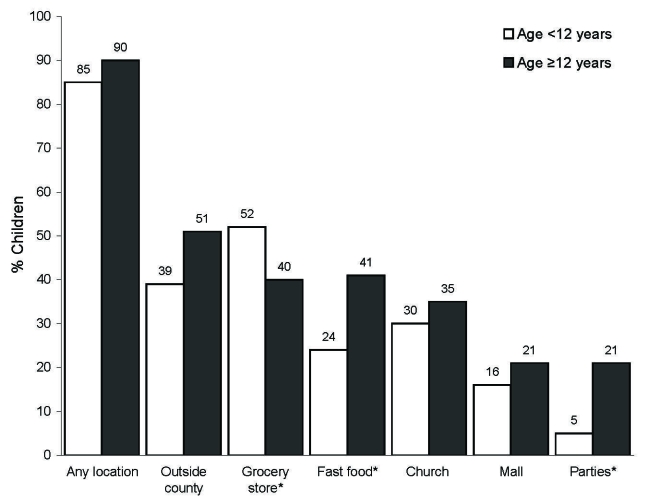
Locations visited by schoolchildren during school closure by age group, controlled for effect of family, Yancey County, North Carolina, 2006. Values above bars are percentages. *p<0.05.

**Figure 3:**
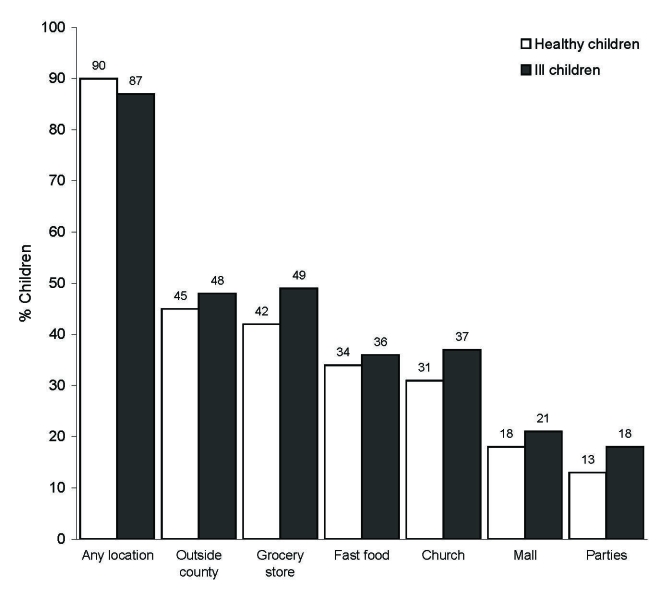
Percentage of ill and healthy schoolchildren visiting various locations during school closure, controlled for effect of family, Yancey County, North Carolina, 2006. Values above bars are percentages. No significant differences were observed (p<0.05).

After we controlled for the effect of family, visiting public locations during the school closure (November 2–12) was commonly reported, with 195 (89%) of 220 children visiting at least 1 public place (Table 3). Overall, 47% of children traveled outside Yancey County at least once during the school closure. Sites children visited during school closure differed by age group and illness status. For example, older children were significantly more likely to go to fast food restaurants and parties (p<0.05; Figure 2). However, they were less likely to go grocery shopping than younger children. No differences were seen between children who were reported as ill at any time from October 23 through November 15 and children who were not ill during that time (Figure 3).

**Table 3:** Locations visited by schoolchildren when schools were closed, controlled for effect of family, Yancey County, North Carolina, 2006

Location visited (N = 220)	No. (%) children
At least 1 public location	195 (89)
Grocery stores	97 (44)
Fast food restaurants	77 (35)
Church services	75 (34)
Mall	42 (19)
Parties or sleepovers	33 (15)
Outside Yancey County	103 (47)

Among adults in surveyed households, 315 (72%) were employed outside the home (Table 4). In 118 (54%) of the 220 households surveyed, all adults in the household were employed outside the home. Of 218 adults living in those 118 homes, only 39 (18%) had occupations that permitted them to work from home. Seventy-six (24%) of the 315 employed adults missed >1 day of work from October 23 through the date of the survey, including 36 (47%) because of their own illness, 18 (24%) to take care of ill family members, and 14 (18%) because of school closure. However, all adults who reported missing work because of school closure were school employees. Among all adults who missed work during the period, the median number of days missed was 3, and days missed ranged from 1 to 14.

**Table 4:** Employment and childcare status of adults and households during school closure, Yancey County, North Carolina, 2006*

Characteristic	Value
Adults employed outside home (N = 438)	315 (72)
Occupations of those employed outside home (N = 315)	
Healthcare	35 (11)
Education	36 (11)
Industry	27 (9)
No. homes where all adults in home employed (N = 220)	118 (54)
Adults who can work from home (in homes where all adults in the house work outside the home, N = 218)	39 (18)
Missed worked from Oct 23 through Nov 15 (N = 315)	76 (24)
Median no. days missed (range)	3 (1–14)
Reason for missed work (N = 76)	
Own illness	36 (47)
Had to take care of ill family members	18 (24)
School closure	14 (18)
Childcare (N = 220)	
Someone home during the day who could provide childcare	167 (76)
Had to make special arrangements	22 (10)
Child had to spend >1 nights away from home for childcare purposes	7 (3)
Had to spend extra money for childcare	2 (1)

*Values are no. (%) unless otherwise indicated.

One hundred sixty-seven (76%) households indicated that someone was regularly available during the day to provide childcare (Table 4). Twenty-two (10%) reported that they had to make special childcare arrangements because of school closure, including enlisting working adult household members, grandparents or other relatives, friends, or nonrelated adults to provide childcare; taking the child to work; having older siblings watch younger children; or using childcare programs. Among responding households, only 7 (3%) had to have their child spend >1 nights outside their household for childcare purposes, and only 2 (1%) reported having to spend extra money ($100 and $150) on childcare arrangements.

A total of 201 (91%) responding households thought the decision to close schools was appropriate (Table 5). Eighty-two (41%) of households that provided a reason for that opinion thought the decision was appropriate to protect the health of the community, 71 (35%) felt there were too many sick children for schools to remain open, 23 (11%) thought it would help protect their child and family, and 8 (4%) thought that schools would be too understaffed if they remained open. Of the 10 (5%) respondents who believed school closure was inappropriate, 4 (40%) thought it could result in lost income, 3 (30%) did not think influenza was in the area, 2 (20%) did not think closure was an effective measure, and 1 (10%) found it too difficult to make childcare arrangements. Overall, 198 (90%) thought they had enough time to prepare for school closures. A total of 180 (84%) felt well prepared and could not think of anything that could have helped them prepare better to deal with closure. Twenty (9%) would have appreciated more time between notification and closing. Several respondents also mentioned that they would have liked to have been warned that influenza was in the area, that children at school were sick, and that schools might be closed as a response to illnesses.

**Table 5:** Household responses to school closure and difficulties obtaining childcare, Yancey County, North Carolina, 2006

Response	No. (%)
Felt closure was appropriate (N = 220)	201 (91)
Reason it was appropriate (N = 201)	
To protect health of the community	82 (41)
Too many sick children	71 (35)
To protect their child and family	23 (11)
Schools would be understaffed	8 (4)
Felt closure was not appropriate (N = 220)	10 (5)
Reason it was not appropriate (N = 10)	
Could result in lost income	4 (40)
Did not think influenza was in the area	3 (30)
Did not think it was an effective measure	2 (20)
Too difficult to make childcare arrangements	1 (10)
Overall preparedness (N = 220)	
Had enough time to prepare for closure	198 (90)
Could not think of anything that would have made them more prepared	180 (84)
Could have used more time	20 (9)

## Discussion

The primary objective of this investigation was to evaluate the response of households to school closure caused by an influenza outbreak. This study found that most adults believed that school closure was appropriate and necessary to slow influenza transmission and protect the health of the community. Second, almost all children visited public areas within the community while schools were closed, despite public health recommendations to avoid large gatherings. Lastly, the effect of school closure on work absenteeism and childcare expenditures appeared to be minimal in this community.

Yancey County is located in the Blue Ridge Mountains, and results obtained there are likely not generalizable to all counties. First, residents in this rural, mountainous county are accustomed to dealing with frequent school closures resulting from adverse weather conditions, particularly winter snowstorms. Families in communities where school closures are infrequent or where extended families are less available to provide childcare might respond differently. Additionally, only 17% of households in this survey were single-adult homes, compared with the national average of 27%. Multiple-adult households might find arranging childcare for schoolchildren during school closures to be less challenging than single-adult homes. Also, the median age of children in this survey was 12 years. Childcare arrangements for older children are likely easier to make than for younger children. Lastly, no adults in this survey reported missing work solely because of school closure, other than those employed by the school. Only 18 (8%) adults from the 220 households in the survey reported missing work to stay with a sick family member. This finding is dissimilar to findings from another study that found that at least 1 adult in 53% of families missed work to care for an ill child because of a winter respiratory illness (*16*). Other studies have also found that epidemics of respiratory illness can cause a substantial number of lost workdays for parents of ill children (*17*,*18*).

Results might also have been different if schools were closed for a longer period or if a more clinically severe strain of influenza were present, causing more hospitalizations or death. Most parents interviewed in the present study did not think that infection would result in hospitalization; only 5 brief hospitalizations were reported. We did not collect quantitative information on the frequency or duration of visits to public places by schoolchildren and did not determine whether these persons visited public places while potentially infectious. Lastly, households with children who attended private school or were home-schooled were not surveyed.

The decision of the local school board to close all 9 schools in Yancey County was primarily motivated by concerns about staffing the schools in the face of high levels of absenteeism. Although the reduction in ILI that occurred after schools were closed is an intriguing finding, results from this investigation cannot be used to assess the effect of school closure on the effect of illness in a community experiencing an influenza outbreak. Influenza outbreak dynamics are relatively poorly understood, and the proportion of children who were susceptible to infection might have decreased below the number required to sustain transmission at approximately the same time schools were closed. The fact that transmission decreased despite many schoolchildren in public gathering places also calls into question the role of reduced contacts among children in ending the outbreak.

Studies that have modeled the effects of NPIs on reducing influenza epidemic size during a pandemic have suggested that closing schools can be effective if implemented early and if the reproductive number (*R*_0_) is low (<1.8) (*19*). For example, in a network-based simulation in which ≈50% of persons were infected, similar to the Asian influenza pandemic of 1957–58, closing schools and keeping those children at home reduced the calculated attack rate by 90% (*20*). Studies of the effects of school closure on respiratory disease rates in schoolchildren have shown mixed results. An investigation in Israel showed that although schools were closed because of a teacher strike, the incidence of respiratory illness diagnosed in children who came to health clinics decreased, as did physician and emergency department visits and purchase of medications (*11*). Rates subsequently increased when schools reopened. However, some (researchers and public health officials) have proposed that school closure in urban areas might have an opposite effect because children released from school can more easily congregate. This effect may have occurred in children from Chicago during the 1918–19 pandemic when rates of influenza among schoolchildren increased during time off from school (*21*). In Connecticut, 3 cities (Bridgeport, Hartford, and New Haven) kept their schools open during the 1918–19 pandemic (*21*) and experienced lower mortality rates than 2 smaller cities (New London and Waterbury) that closed schools.

We cannot assess the effect of influenza vaccination on the course of the influenza outbreak in Yancey County. Large-scale vaccination programs began in the county during late October. Although not known at the time of these programs, the influenza B strain contained in the 2006–2007 vaccine (Victoria lineage) was not a good match to the circulating Yamagata lineage influenza B viruses in this outbreak (*22*). However, only a few influenza virus isolates were antigenically characterized at the Centers for Disease Control and Prevention (CDC) in Atlanta, and we do not know that the isolates tested were representative of those circulating in the area at the time. Influenza A viruses were found through CDC’s sentinel surveillance systems to be circulating in other counties concurrently. It is possible that other viruses were co-circulating in Yancey County and were not detected.

The effectiveness of closing schools to reduce transmission of common infectious diseases such as influenza is not well studied (*12*). In addition, data on school closures in response to infectious disease outbreaks in general are not regularly collected in the United States. Additional studies to assess these actions will be of interest to local public health officials and school administrators who make decisions about keeping schools open during explosive but self-limited outbreaks with high attack rates among schoolchildren, such as those commonly caused by seasonal influenza or norovirus. Results from such studies will also be helpful in planning and implementing community mitigation strategies for disease outbreaks whose community impact might be severe, such as pandemic influenza.

This investigation provides insight into how households with school-age children in a small rural community responded to a brief school closure precipitated by a seasonal influenza outbreak. Overall, respondents to the survey indicated that the community was in favor of closing schools as a way to deal with high levels of student and staff absenteeism and potentially to reduce transmission. Parents reported few problems in coping with the school closure and did not miss work to provide childcare. However, many students visited public areas during school closure. Plans for pandemic influenza responses should address the potential for transmission in public areas during school closure.
